# Genetic Mechanisms Highlight Shared Pathways for the Pathogenesis of Polygenic Type 1 Diabetes and Monogenic Autoimmune Diabetes

**DOI:** 10.1007/s11892-019-1141-6

**Published:** 2019-03-19

**Authors:** Matthew B. Johnson, Karen Cerosaletti, Sarah E. Flanagan, Jane H. Buckner

**Affiliations:** 1 Institute of Biomedical and Clinical Science, University of Exeter Medical School, Exeter, UK; 2 Translational Research Program, Benaroya Research Institute at Virginia Mason, Seattle, WA USA

**Keywords:** Monogenic diabetes, Polygenic risk, Autoimmunity, Immune tolerance

## Abstract

**Purpose of Review:**

To highlight pathways important for the development of autoimmune diabetes by investigating shared mechanisms of disease in polygenic and monogenic diabetes.

**Recent Findings:**

Genome-wide association studies have identified 57 genetic risk loci for type 1 diabetes. Progress has been made in unravelling the mechanistic effects of some of these variants, providing key insights into the pathogenesis of type 1 diabetes. Seven monogenic disorders have also been described where diabetes features as part of an autoimmune syndrome. Studying these genes in relation to polygenic risk loci provides a unique opportunity to dissect pathways important for the development of immune-mediated diabetes.

**Summary:**

Monogenic autoimmune diabetes can result from the dysregulation of multiple pathways suggesting that small effects on many immune processes are required to drive the autoimmune attack on pancreatic beta cells in polygenic type 1 diabetes. A breakdown in central and peripheral immune tolerance is a common theme in the genetic mechanisms of both monogenic and polygenic disease which highlights the importance of these checkpoints in the development and treatment of islet autoimmunity.

## Introduction

Type 1 diabetes (T1D) is a complex disease that arises in the context of genetic risk and environmental triggers. Together, these alter immune pathways, resulting in the destruction of insulin-producing pancreatic beta cells. Despite the recognition of an autoimmune aetiology in T1D over 40 years ago [1], knowledge of the triggers and underlying mechanisms of T1D remain incomplete. Efforts to elucidate the cellular pathology of T1D in humans have been hampered by a limited ability to access human pancreatic tissue for direct examination and the immunologic and clinical heterogeneity of the disease.

Genetic variation at the HLA region on chromosome 6p21 confers the greatest polygenic risk for the development of T1D. A further 56 non-HLA loci have also been identified but these confer a lower risk for development of the disease [2••, 3]. Many of these genetic loci are associated with additional autoimmune diseases (Table 1), including specific HLA alleles (Table 2). Seven monogenic conditions are also known to cause autoimmune diabetes that is clinically indistinguishable from T1D. In these patients, diabetes usually presents as part of a syndrome of multiple autoimmunity [9].

**Table 1:** Non-HLA loci associated with both type 1 diabetes (T1D) and additional autoimmune diseases

Gene(s)	dbSNP ID	Diseases associated
*PHTF1*, *PTPN22*	rs6679677, rs2476601	ATD, CD, JIA, RA, SLE, T1D, AA, VIT
*IL10*	rs3024493, rs3024505	CD, SLE, T1D, UC, IBD
*IFIH1*	rs1990760, rs35667974, rs2111485	PSO, SLE, T1D, UC, IBD, VIT
*CTLA4*	rs3087243, rs11571316	ATD, CD, RA, T1D
*AFF3*	rs9653442	RA, T1D
*CCR5*	rs113010081	CEL, T1D, UC
*IL21*, *ADAD1*, *IL2*	rs17388568, rs4505848, rs75793288, rs6827756	CEL, CD, T1D, UC
*IL7R*	rs11954020, rs6897932	T1D, MS
*BACH2*	rs11755527, rs597325, rs72928038	ATD, MS, RA, T1D
*TNFAIP3*	rs6920220	RA, SLE, T1D, UC, IBD
NA	rs6916742, rs9272346, rs9268645	CEL, T1D
*TAGAP*	rs1738074	CEL, MS, T1D
*RBM17*, *IL2RA*	rs2104286, rs61839660, rs7090530, rs10795791, rs12251307, rs41295121	MS, RA, T1D
*BAD*	rs694739	CD, MS, T1D, AA
*IKZF4*, *DGKA*, *ERBB3*	rs11171739, rs705704, rs2292239, rs11171710, rs705705	T1D, AA
*NAA25*, *SH2B3*	rs3184504, rs653178, rs17696736	CEL, CD, JIA, PBC, RA, T1D, AA, PSC, VIT
NA	rs911263	PBC, T1D
*CTSH*	rs3825932, rs12148472, rs34593439	CEL, T1D, NAR
*RASGRP1*	rs12908309, rs72727394	CD, T1D
*IL27*	rs4788084, rs9924471, rs151234	ANS, CD, T1D, IBD
*DEXI*, *CLEC16A*	rs12927355, rs193778, rs12708716	MS, PBS, T1D
NA	rs7221109	T1D, UC
*ORMDL3*, *GSDMB*	rs2290400, rs12453507	CD, T1D, UC, IBD
*CD226*	rs1615504, rs763361	MS, T1D
*PTPN2*	rs2542151, rs1893217	CEL, CD, T1D, UC, IBD
*FUT2*	rs516246, rs602662	CD, T1D, IBD
*TYK2*	rs12720356, rs34536443	CD, JIA, MS, PBC, PSO, RA, T1D, IBD
*UBASH3A*	rs11203202, rs11203203	RA, T1D, VIT

Data from Immunobase.org (https://www.immunobase.org/) [4]

*AA* alopecia areata, *ANS* ankylosing spondylitis, *ATD* autoimmune thyroid disease, *CD* Crohn’s disease, *CEL* Coeliac disease, *IBD* inflammatory bowel disease, *JIA* juvenile idiopathic arthritis, *MS* multiple sclerosis, *NAR* narcolepsy, *PBC* primary biliary cirrhosis, *PSC* primary sclerosing cholangitis, *PSO* psoriasis, *RA* rheumatoid arthritis, *SC* scleroderma, *SJ* Sjogren’s syndrome, *SLE* systemic lupus erythematosus, *UC* ulcerative colitis, *VIT* Vitiligo, *NA* not applicable

**Table 2:** Shared risk of autoimmune diseases conferred by the HLA DR3 (DRB1*0301-DQA1*0501-DQB1*0201) and DR4 (DRB1*0401-DQA1*0301)

HLA allele	Disorder	Odd’s ratio	Reference
DR3	Type 1 diabetes	3.64	[5] Erlich et al. 2008 Diabetes
	Coeliac disease	2.09	[6] Liu et al. 2014 NEJM
	Hypothyroidism	2.53	[7] Zamani et al. 2000 AJMG
DR4	Type 1 diabetes	7.03	[5] - Erlich et al. 2008 Diabetes
	Multiple sclerosis	1.63	[4] Andlauer et al. 2016 Sci Adv
	Rheumatoid arthritis	2.88	[8] Stahl et al. 2010 Nat Genet

Odds ratios are provided for individuals carrying a single copy of each allele

In this review, we will discuss how studying monogenic autoimmune disease has informed our understanding of mechanisms that contribute to polygenic disease. We will highlight pathways which are shared in the pathogenesis of T1D and other organ specific autoimmunity, focussing on those that have a role in both monogenic forms of autoimmune diabetes and polygenic T1D.

## A Breach in Immune Tolerance Is Key to the Development of Autoimmunity

### Loss of Central Tolerance

Central tolerance is the process of removing self-reactive T or B cells during their development thus preventing them from targeting normal tissues in the periphery [10]. This process occurs in the thymus for T cells and requires that the T cell receptor (TCR) of a developing T cell is able to bind human leukocyte antigen (HLA) molecules (those reacting to class I HLA go on to form CD8+ T cells, while class II form CD4+ T cells), while limiting the development of T cells with receptors that bind self-peptides in the context of HLA with high affinity. When central tolerance fails, autoimmunity can arise through the release and subsequent action of self-reactive T cells.

### Loss of Central Tolerance in Monogenic Disease

The Autoimmune Regulator gene, ***AIRE***, regulates the ectopic expression of self-peptides within the thymus in order to expose naïve T cells to these peptides during development [11]. Loss of function mutations in *AIRE* (either recessive or dominantly inherited) cause autoimmune polyendocrine syndrome type 1 (APS1, also known as autoimmune polyendocrinopathy-candidiasis-ectodermal dystrophy, APECED) by reducing or removing this function of AIRE in the thymus [12]. This allows high affinity autoreactive T cells to escape the thymus. Clinically, APS1 is highly variable but is usually characterised by chronic mucocutaneous candidiasis, adrenal insufficiency and autoimmune hypoparathyroidism. Approximately 13% of individuals develop autoimmune diabetes by 30 years of age [13].

### T1D Genetic Risk Loci Involved in Central Tolerance

Variation in the insulin gene (***INS***) is linked to the development of T1D and is thought to result in a failure of central tolerance. The T1D-associated polymorphic variant is considered to be a variable number of tandem repeats (VNTR), located in the promoter of the *INS* gene to which AIRE binds, regulating *INS* RNA expression in the thymus [14–19]. VNTR variants of smaller size (class I VNTRs) are associated with increased T1D risk and lower INS mRNA expression in the thymus, allowing escape of insulin autoreactive CD4 T cells into the periphery during T cell development due to fewer insulin peptide-HLA class II interactions. Conversely, insulin autoreactive T cells are predicted to be deleted in individuals carrying the protective *INS* variants (Class III VNTRs) which drives higher levels of *INS* expression in the thymus [16]. In keeping with a failure in central tolerance, insulin autoreactive CD4 T cells are present at a higher frequency in the peripheral blood of T1D subjects carrying the *INS* susceptibility variants, whereas individuals with protective alleles have barely detectable levels of insulin autoreactive CD4 T cells [20].

A failure in central tolerance may also contribute to the association of **HLA class II genes** to T1D. Although the mechanism is not completely understood, evidence points to low affinity interactions between class II DQ8 molecules and islet peptides, which may result in failed deletion of islet autoreactive CD4 T cells [21, 22]. Another T1D-associated gene, ***PTPN22***, has been linked with failures in both central and peripheral tolerance of T and B cells [23, 24]. A failure of B cell tolerance may be due in part to altered B cell receptor signalling in the presence of the risk variant p.R620W in *PTPN22*, allowing autoreactive B cells to escape central and peripheral tolerance checkpoints [25]. Although T1D is considered a T cell–mediated disease, B cell pancreatic infiltrate is present in many childhood onset T1D cases [26, 27] and anti-CD20 B cell depleting therapy temporarily slowed disease progression in established T1D [28], indicating a role for B cells in T1D pathogenesis.

### Reduced Peripheral Tolerance

Central tolerance is an imperfect process, and as such peripheral tolerance exists to regulate self-reactive cells that escape thymic negative selection. Regulatory T cells (Tregs), a specialised subset of CD4+ T cells, are critical for peripheral tolerance [29, 30]. Tregs suppress conventional T cell (CD8+ and CD4+) activation, proliferation and cytokine production after an immune response to prevent collateral damage to tissues once a pathogen has been removed. There is also growing evidence supporting suppression of B and dendritic cells by Tregs [31, 32].

### Reduced Peripheral Tolerance in Monogenic Disease

Reduced function or number of Tregs has been implicated in the disease mechanism of several monogenic autoimmune disorders. Immunodysregulation, polyendocrinopathy, enteropathy, X-linked (IPEX) syndrome highlights the requirement of Tregs to restrain autoimmunity. IPEX syndrome, which often proves fatal in early life, results from hemizygous mutations in the ***FOXP3*** gene, a key regulator of Treg development [33]. The syndrome typically presents in the neonatal period, with > 90% of affected boys having severe protein losing enteropathy and ~ 80% developing autoimmune diabetes. Additional autoimmune diseases can develop including severe atopic dermatitis (70%) and autoimmune hypothyroidism (35%) [34].

Individuals with recessively inherited ***IL2RA*** mutations develop immunodeficiency 31C syndrome [35], which is similar to IPEX syndrome (enteropathy, hypothyroidism and severe eczema) and can include neonatal-onset autoimmune diabetes [36]. *IL2RA* encodes CD25, a subunit of the IL-2 receptor which is constitutively and highly expressed on regulatory T cells facilitating their recruitment and suppressive ability. IL-2 is a key signalling molecule involved in regulating the immune system and induces *FOXP3* expression; therefore, loss of the IL-2 receptor on T cells reduces *FOXP3* expression and Treg development [37]. As in IPEX, mutations that lead to a loss of CD25 expression result in a reduced Treg compartment, promoting autoimmunity through the failure of peripheral tolerance [38].

Heterozygous mutations in ***CTLA4*** cause autoimmune lymphoproliferative syndrome which can include autoimmune diabetes [39–41]. CTLA4 is constitutively expressed by Tregs and can also be expressed by CD4+ and CD8+ effector T cells where it functions as a potent suppressive receptor molecule, by preventing co-activation of T effector cells via CD28 [42]. It mediates inhibition in effector T cells by competing with CD28 for binding to CD80/CD86 on antigen presenting cells (APC) but may also inhibit T cell receptor signalling [31, 43, 44]. Tregs from individuals with dominantly inherited *CTLA4* mutations show reduced expression of CTLA4, FOXP3 and IL2RA [39].

Recessively inherited mutations in ***LRBA*** cause common variable immunodeficiency-8 (CVID8) with autoimmunity [45]. This includes extremely young onset of haematological autoimmune disorders (80%), enteropathy (70%) and autoimmune diabetes (30%) [46]. LRBA plays an essential role in the post-translational regulation and trafficking of CTLA4 (see above), whereby it prevents lysosomal degradation of CTLA4 containing vesicles [47•]. Interestingly, studies have identified six individuals with LRBA mutations who had a reduced number of Tregs [48] and an individual with normal cell-surface CTLA4 expression [49]. In the latter patient, there was increased Th17 cell activity (measured by IL-17 production) suggesting that the disease could also be mediated through effector cells.

Gain of function (GOF) mutations in ***STAT3***, which links extracellular cytokine signals to gene expression, cause infancy-onset multiple autoimmune disease [50]. These mutations cause haematological autoimmune disorders (70%), enteropathies (50%) and autoimmune diabetes in ~ 30% of individuals which often presents in the neonatal period. Some patients present with similar features to autoimmune lymphoproliferative syndrome (ALPS) [51, 52]. STAT3 is involved in multiple signalling pathways that influence the fate of CD4 T cells, enhancing development of Th17 and T follicular helper cells, while blocking the development and survival of regulatory T cells. Tregs are numerically and functionally reduced in most individuals with GOF *STAT3* mutations, while Th17 cells may be normal, reduced or increased [53].

### T1D Genetic Risk Loci Involved in Peripheral Tolerance

Genetic variants in genes that function in the IL-2 pathway are associated with T1D, including ***IL2RA*** (described above) and ***PTPN2*** which encodes a non-receptor tyrosine phosphatase that regulates IL-2 signalling [2••, 54, 55]. The most highly associated *IL2RA* single nucleotide polymorphism (SNP), rs61839660, is non-coding and located in an enhancer region in intron 1 of the gene. The enhancer binds multiple transcription factors and interacts with other regulatory elements in the locus in primary CD4 T cells, but only in response to T cell stimulation [56••]. The presence of the T1D risk allele at the enhancer resulted in enhanced CD25 (IL2RA) upregulation in CD4 conventional T cells but not Tregs in a knock-in mouse model [56••], indicating that this SNP primarily impacts CD4 conventional T cells.

Alternatively, several other *IL2RA* SNPs have been associated with decreased CD25 expression on CD4 conventional T cells and Tregs, and increased levels of soluble CD25 with the risk alleles, revealing the complexity of the *IL2RA* locus [57–59]. Functionally, this correlates with reduced IL-2 signalling and diminished Treg fitness and suppressive function [58, 59]. The T1D-associated SNPs in the *PTPN2* gene are also non-coding and have been correlated with decreased *PTPN2* RNA levels and reduced IL-2 signalling in genotyped healthy control subjects and longstanding T1D patients [60, 61]. The *PTPN2* T1D risk allele was also associated with decreased FOXP3 expression in activated CD4 T cells [60]. The effects of these *IL2RA* and *PTPN2* T1D risk alleles on IL-2 signalling are independent but additive, both potentially contributing to reduced peripheral tolerance through effects on Tregs [58]. An additional T1D-associated SNP is located in the ***IL2-IL21*** intergenic region, although the impact of this SNP has not been evaluated yet [2••].

A non-coding SNP rs3087243 located 3′ of the ***CTLA4*** gene has been associated with T1D, as well as other autoimmune diseases [2••] (Table 1). How the *CTLA4* rs3087243 SNP affects CTLA4 function is not completely understood. Initial studies indicated that the rs3087243 variant affected *CTLA4* alternative splicing, resulting in lower levels of a soluble CTLA4 isoform in CD4 T cells carrying the T1D susceptibility allele [62]. However, this finding was not replicated in a subsequent study [63]. More recently the rs3087243 SNP was shown to be in high linkage disequilibrium with an (AT)_n_ dinucleotide repeat in the 3′ untranslated region of *CTLA4*, with the T1D susceptibility allele associated with longer (AT)_n_ repeat length compared with the non-risk allele [64]. Human islet autoreactive T cell lines with longer (AT)_n_ repeats expressed lower levels of *CTLA4* RNA and protein relative to T cell lines with shorter repeats, and longer (AT)_n_ repeats destabilised a GFP reporter expressed in Jurkat T cells [64]. Confirmation of these findings in rs3087243 genotyped peripheral blood CD4 T cells and elucidation of corresponding functional phenotypes will clarify the mechanism of the T1D association with *CTLA4*.

## Defects in Interferon Signalling Pathways

Interferons (IFNs) are cytokines released by mononuclear cells in response to the presence of pathogens and tumours that modulate the immune system. They have a role in the regulation of immune responses, activating natural killer cells and macrophages and upregulating antigen presentation by HLA molecules [65]. Type 1 IFNs (IFN-1) are central to the anti-viral response, yet the presence of an IFN signature is well described in multiple autoimmune diseases including T1D [66, 67]. Perturbed IFN signalling is associated with systemic lupus erythematosus, a systemic autoimmune condition directed against ubiquitous proteins such as those within the cell nucleus [68]. Indeed, an IFN signature consisting of expression levels of type 1 IFN-responsive genes correlates with the severity of disease in SLE [69]. The mechanistic role of IFN signalling in the induction of organ specific autoimmunity such as T1D remains unclear although evidence is mounting that they may induce increased autoantigen presentation by islet cells and thus increase recognition and activation of effector T cells [70]. Studies on deceased T1D patients’ pancreata have shown increased expression of IFN alpha [71, 72]; however, whether this was secondary to, for example, viral infection of the islets, remains unknown. The role of IFNs in organ specific autoimmunity is further supported by the triggering of autoimmune disease, including diabetes, in patients undergoing interferon treatment for malignancies [73, 74].

### Defects in IFN Signalling in Monogenic Disease

Patients with dominant GOF mutations in ***STAT1*** present with chronic mucocutaneous candidiasis and lower respiratory tract infections with a subset of patients developing severe organ-specific autoimmunity including T1D (4% of patients) [75]. STAT1 responds to multiple cytokines to translate extracellular signals to gene transcription. IFN alpha induces the transcription of numerous targets via the JAK-STAT pathway increasing the proliferation of immune cells, as well as augmenting other immune processes such as antibody production. Increased activity of STAT1, as a result of impaired nuclear de-phosphorylation, is proposed to lead to autoimmunity by increasing IFN alpha signalling.

### T1D Genetic Risk Loci Involved in IFN Signalling

Two coding variants in genes within the interferons pathway, ***IFIH1*** **A946T** and ***TYK2*** **P1104A**, support a role of IFN-1 in the development of T1D [2••, 76].

IFIH1 (interferon-induced helicase C-domain-containing protein 1, also known as MDA5) is a component of the innate response to RNA viruses. Upon recognition of double-stranded RNA, IFIH1 undergoes a conformational change that result in a series of signalling events leading to transcription of genes encoding IFN-1 and interferon-stimulated genes (ISGs). Rare GOF missense mutations in *IFIH1* are described which result in interferonopathies [77]. A rare loss of function mutation in the *IFIH1* gene is associated with protection from T1D [78]. The common variant rs1990760, a non-synonymous coding variant in *IFIH1*(A946T), is associated with risk for T1D [76] as well as other autoimmune diseases including SLE [79]. This variant results in enhanced basal expression of IFN-1 and improved response to viral challenge indicating that the *IFIH1* risk variant 946T is a gain-of-function variant that is triggered by RNA self-ligands as well as viral infection [80]. This variant has likely been selected in the population as it provides an advantage in the setting of viral infection, despite the fact that it also promotes the risk of autoimmunity.

*TYK2* encodes a JAK family kinase that functions to mediate proximal IFN-1-, IL-12- and IL-23-dependent signals [81, 82]. TYK2 deficient patients exhibit susceptibility to viral infections and impairment in cellular responses to IFN-1, as well as mycobacterial infections consistent with an impaired response to IL-12 and IL-23. A missense change within the kinase domain of TYK2, with substitution of alanine for a conserved proline (TYK2-P1104A) is associated with a lack of catalytic activity and protection for T1D [2••, 83]. The protective *TYK2* 1104A allele results in impaired cellular responses to IFN-1, IL-12 and IL-23 and notably leads to a decrease in the induction of experimental autoimmune encephalomyelitis in murine models and a striking decrease in IL-17/IFN-γ positive CD4 T cells [83, 84]. In humans carrying the TYK2-1104A allele, IFN-1 receptor signalling is decreased, and alterations in memory B cell populations and a decrease in Tfh cells are seen in the peripheral blood [84, 85]. Similar to *TYK2* deficient patients, individuals homozygous for the *TYK2* 1104A variant have an increased frequency of tuberculosis [83]. The incidence of the *TYK2* 1104A allele has decreased over the past 4000 years in Europeans, consistent with negative selection of this allele by tuberculosis infection [83]. Thus, the T1D risk allele *TYK2* P1104 is increasing in frequency in European populations. Taken together, these studies suggest that alterations in IFN signalling in T1D may play a role in disease, but in the case of the *TYK2* variant, this is in the broader context of other cytokine responses.

## Conclusions

The finding that monogenic autoimmune disease can result from the dysregulation of multiple immune pathways suggests that small effects on multiple processes may be required to drive the autoimmune attack on pancreatic beta cells in polygenic T1D. A common theme in the genetic pathways identified in diabetes and other autoimmunity is the breakdown of immune tolerance; however, the part of the pathway perturbed can be different. Monogenic disorders include those which disrupt central tolerance (*AIRE*), or impact the development (*FOXP3*, *STAT3*) or function (*IL2RA*, *LRBA*, *CTLA4*) of regulatory T cells required for the maintenance of peripheral tolerance. The risk alleles identified by genome-wide association studies mirror this, with effects on the ectopic expression of insulin, reducing central tolerance (e.g. *INS* VNTR), and reduced expression of genes essential for the maintenance of peripheral tolerance also identified (e.g. *IL2RA*, *PTPN2*, *CTLA4*).

Defects in interferon signalling are increasingly recognised as being involved in multiple autoimmune diseases. While diabetes is a rare feature of monogenic interferonopathies, two loci within the IFN pathway (*IFIH*-1 and *TYK2*) have been robustly associated with polygenic T1D. Furthermore, the presence of an IFN signature in some patients with T1D, increased expression of IFN in pancreata from deceased T1D patients and mounting evidence of viral involvement in some T1D cases highlight that defects in IFN signalling may be an important contributor to the pathophysiology of T1D. Further molecular characterisation of the mechanisms by which common risk loci that impart risk for T1D and of genes causing monogenic autoimmunity will be the key to detecting the pathways that underlie this shared aetiology.
